# Concise Syntheses of Trifluoromethylated Cyclic and Acyclic Analogues of cADPR

**DOI:** 10.3390/molecules15128689

**Published:** 2010-11-30

**Authors:** Xiangchen Huang, Min Dong, Jian Liu, Kehui Zhang, Zhenjun Yang, Liangren Zhang, Lihe Zhang

**Affiliations:** State Key Laboratory of Natural and Biomimetic Drugs, School of Pharmaceutical Sciences, Peking University, Beijing 100191, China

**Keywords:** cADPR analogue, acyclic cADPR analogue, trifluoromethylation, synthesis

## Abstract

A novel trifluoromethylated analogue of cADPR, 8-CF_3_-cIDPDE (**5**) was designed and synthesized *via* construction of *N^1^,N^9^*-disubstituted hypoxanthine, trifluoromethylation and intramolecular condensation. A series of acyclic analogues of cADPR were also designed and synthesized. These compounds could be useful molecules for studying the structure-activity relationship of cADPR analogues and exploring the cADPR/RyR Ca^2+^ signalling system.

## 1. Introduction

**Figure 1:**
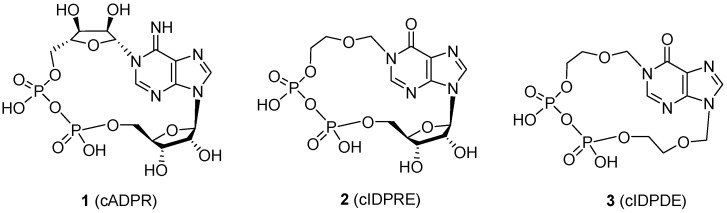
Structures of cADPR and its analogues.

Cyclic adenosine diphosphate ribose (cADPR, **1**, Figure 1), isolated from sea urchin eggs [1], is a metabolite of β-nicotinamide adenine dinucleotide (NAD^+^). It has been proved that cADPR is a signalling molecule, which regulates calcium mobilization via ryanodine receptor (RyR) in a wide variety of Ca^2+^-dependent cellular responses such as fertilization, secretion, contraction, proliferation and so on [2]. Since the discovery of cADPR, numerous works have been done on the synthesis of cADPR analogues to search for agonists or antagonists of cADPR/RyR Ca^2+^ signalling system [3,4,5].

In our previous work, a series of cADPR analogues in which the southern and/or northern ribose was replaced by an ether chain were synthesized [6,7]. Most of those compounds, such as cIDPRE (**2)** and cIDPDE (**3**), are membrane permeate agonists in Jurkat T cells. 

**Figure 2:**
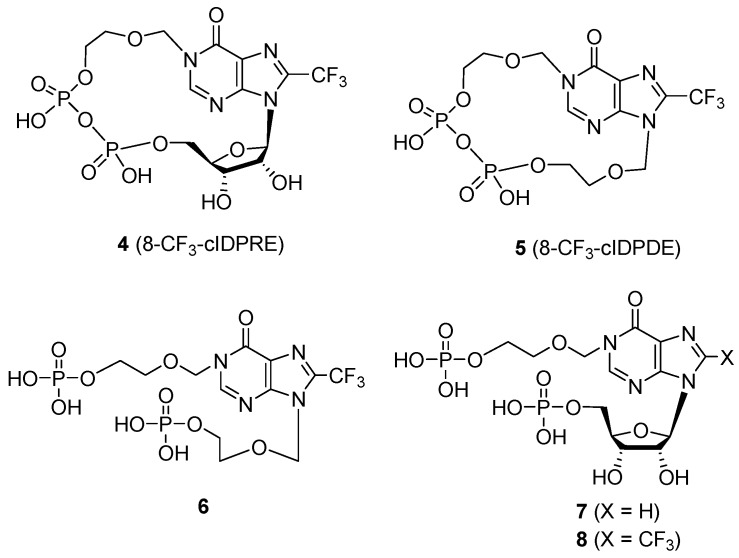
Structures of compounds **4-8**.

Moreover, it was found that those agonists antagonize the hydrolysis of CD38. Substitution at C-8 of purine affects the agonistic activity of cADPR analogues. For example, 8-Br or 8-Cl substituted cIDPRE loses activity; however, the activity is retained for 8-N_3_ or 8-NH_2_ substituted cIDPRE. These results indicate that the effect of substitution at 8-position depends on the property of the substituent group. The trifluoromethyl group, possessing high electronegativity and lipophilicity, usually alters considerably the overall charge distribution and enhances the membrane permeability of molecules. Since the trifluoromethyl group imparts a variety of special physical and chemical properties to molecules, a number of trifluoromethylated compounds exhibit enhanced biological activity [8]. Taking these points into account, we synthesized 8-CF_3_-cIDPRE (**4**, Figure 2). We found that this compound was also a membrane permeate calcium agonist in Jurkat T cells [9]. In this study, the trifluoromethyl group is introduced to cIDPDE (8-CF_3_-cIDPDE, **5**, Figure 2). This compound provides a complementary agent for understanding the effect of 8-substitution on calcium signalling property.

cADPR can be hydrolyzed either *in vivo* or *in vitro* [10,11]. The cyclic pyrophosphate moiety, as one of the most vulnerable linkages in cADPR, can be hydrolyzed by Mn^2+^-dependent ADP-ribose/CDP-alcohol pyrophospatase to afford the bisphosphate metabolite [12]. Recently, a series of acyclic analogues of cADPR, in which the pyrophosphate moiety is cleaved to give a bisphosphate, have been synthesized [13]. The primary pharmacological research revealed that some of them could inhibit cIDPRE-induced Ca^2+^ release. To further explore the Ca^2+^-modulating activities of this novel class of cADPR mimics and their mechanism further, we have designed and synthesized acyclic analogues of cIDPRE and the trifluoromethylated analogues **6**-**8** (Figure 2).

## 2. Results and Discussion

### 2.1. Synthesis of 8-CF_3_-cIDPDE (**5**)

**Scheme 1:**
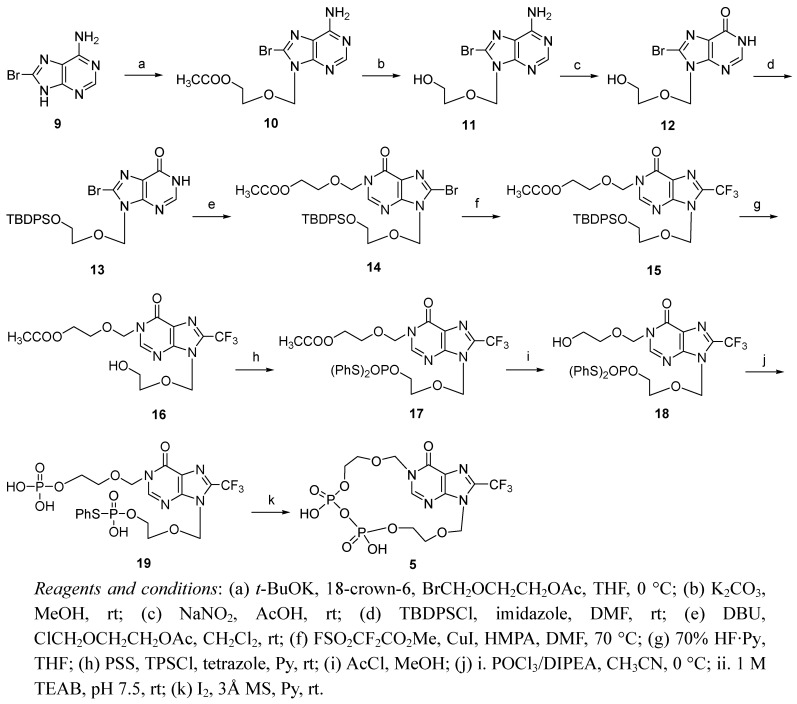
Synthesis of 8-CF_3_-cIDPDE (**5**).

The synthesis of 8-CF_3_-cIDPDE is summarized in Scheme 1. Starting from 8-bromoadenine [14], *N^9^*-substitution was carried out with (2-acetoxyethoxy)methyl bromide [15] in the presence of potassium *tert*-butoxide (*t*-BuOK) and 18-crown-6 [16] to afford **10** in 44% yield. It is noteworthy that when (2-acetoxyethoxy)methyl chloride was employed instead, replacement of the 8-bromo group with a chlorine atom was observed. The structure of compound **10** was confirmed by ^1^H-NMR, ^13^C-NMR, HMBC and HR-ESI-MS spectra. In the HMBC spectrum of **10**, the correlation between H-1′ of the ether chain and C-4 and C-8 of adenine base were observed, which verified that the substitution was on N-9. 

**Figure 3:**
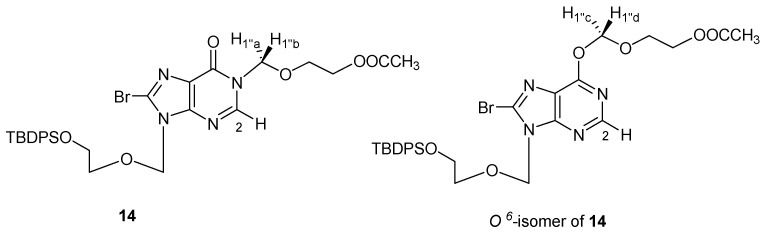
Structures of **14 and its** *O^6^*-isomer.

Deacetylation of **10** with K_2_CO_3_/MeOH gave compound **11**, and after diazotization, and protection of the 5′-hydroxyl group with a *tert*-butyldiphenylsilyl (TBDPS) group, **13** was obtained. An *N*^1^-substitution was carried out on compound **13** with (2-acetoxyethoxy)methyl chloride in the presence of excess 1,8-diazabicyclo[5.4.0]undec-7-ene (DBU) to afford **14** in 61% yield. Since both of the *N*^1^ and the *O^6^* have nucleophilicity, the *N*^1^-isomer and *O*^6^-isomer were obtained (Figure 3). The structure of **14** was confirmed by ^1^H-NMR, ^13^C-NMR, HMBC and HR-ESI-MS spectra. In the HMBC spectrum of **14**, the correlation between H-1′′of the northern ether chain and C-2 of hypoxanthine base, and that between C-1′′ of the northern ether chain and H-2 of hypoxanthine base were both observed, which were similar to that of *N^1^*-isomer. Corresponding correlations were not found in the HMBC spectrum of the *O*^6^-substituted side product.

The unstable glycosylic bond in nucleosides is sensitive to certain conditions, which causes great difficulties in the trifluoromethylation of nucleosides. In our previous work, methyl fluorosulphonyl- difluoroacetate/copper iodide (FSO_2_CF_2_CO_2_Me/CuI) [17] was initially applied to the synthesis of 8-CF_3_-purine nucleosides [9]. Adopting this strategy, trifluoromethylation of **14** was achieved successfully, and optimization of this reaction was carried out (Table 1). Under the optimal reaction conditions, **15** was obtained in 42% yield, and **14** was recovered in 17% yield. Interestingly, compound **16** was also obtained in a yield of 14%. It is known that the tert-butyldimethylsilyl (TBDMS) group and TBDPS group could be removed by tetrabutylammonium fluoride/tetrahydrofuran (TBAF/THF), potassium fluoride and other agents containing fluoride [18]. Accordingly, we deduced it was the fluoride ion generated in the process of trifluoromethylation [17] that facilitated the removal of the 5′-*O*-TBDPS group. The trifluoromethylated product **15** was characterized by ^1^H-NMR,^13^C-NMR, ^19^F-NMR and HR-ESI-MS spectra. In the ^13^C-NMR spectrum of compound **15**, signals of the CF_3_ group and C-8 were spilt into two quartets, with ^1^*J*_CF_ = 270 Hz and ^2^*J*_CF_ = 41 Hz, respectively, and the singlet at −63.358 ppm was observed in the ^19^F-NMR spectrum. These data strongly support the incorporation of the trifluoromethyl group. 

The 5′-*O*-TBDPS group in compound **15** was removed by employing 70% HF·pyridine [19]. The strong electronegativity of trifluoromethyl group at C-8 of hypoxanthine makes the glycosylic bond rather sensitive to acid conditions. Hence, 70% HF·pyridine was added dropwise to the reaction mixture at −20 °C. Compound **16** was successfully converted to **17** by the reaction with *S,S*-diphenylphosphorodithioate (PSS) [20] in the presence of triisopropylbenzenesulfonyl chloride (TPSCl) and tetrazole in pyridine, in a yield of 79%. Considering the instability of phenylthio group under basic conditions [21], acetyl chloride in methanol (AcCl/MeOH) [22] was applied to the deacetylation of **17**. When 1.2 equivalent of AcCl was utilized, compound **17** was successfully converted to **18**. Phosphorylation of the 5′′-hydroxyl in **18** was carried out in the presence of excess POCl_3_ and *N*,*N*-diisopropylethylamine (DIPEA) at 0 °C. After being stirred for 14 h, the mixture was treated with 1 M triethylammonium bicarbonate (TEAB) for 6 h at room temperature [23], which facilitated the semi-deprotection of the *S,S*-diphenylphosphate. Purified by high performance liquid chromatography (HPLC), compound **19** was obtained as its triethylammonium salt. 

Following the Matsuda strategy [24], with excess I_2_ and 3Å molecular sieves as promoters, the intramolecular cyclization was performed in pyridine by adding a solution of compound **19** slowly over 20 h utilizing a syringe pump. Purification by HPLC afforded cyclic product **5** as its triethylammonium salt in 71% yield, which was characterized by ^1^H-NMR, ^19^F-NMR, ^31^P-NMR and HR-ESI-MS spectra. 

### 2.2. Syntheses of Compounds **6-8**

**Scheme 2:**
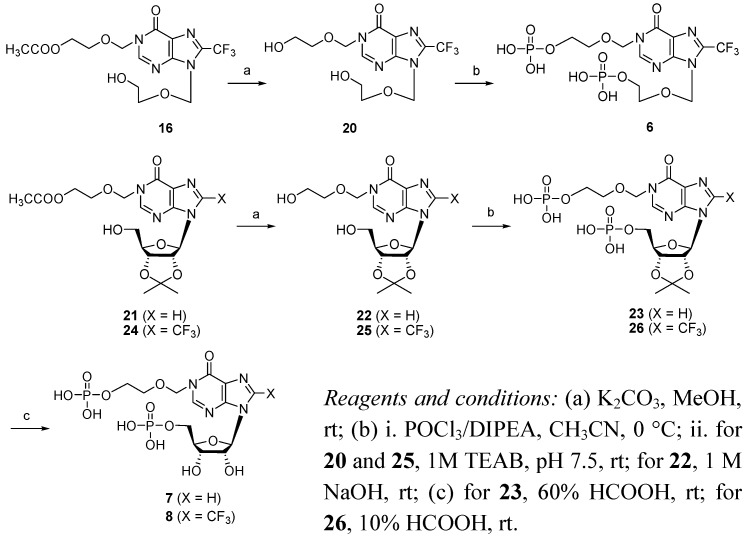
Syntheses of compounds **6-8**.

Deacetylation of **16** with K_2_CO_3_/MeOH afforded compound **20** (Scheme 2), then both of the free hydroxyl groups in **20** were phosphorylated by employing POCl_3_/DIPEA in CH_3_CN at 0 °C for 16 h, followed by the treatment with 1 M TEAB for 6 h. Purified by HPLC, the target molecule **6** was obtained as its triethylammonium salt in 62% yield for two steps.

Compound **23** was synthesized from **21** [6] in a yield of 71% for two steps by a similar method as used for the preparation of **6**. After removing the 2′,3′-*O*-isopropylidene group using 60% HCOOH solution, compound **7** was obtained as its triethylammonium salt in 85% yield. Starting from compound **24 [9]**, **26** was synthesized by a similar procedure. Considering the sensitivity of 8-CF_3_-purine nucleosides to acid conditions, we performed the deprotection of **26** by employing 10% rather than 60% HCOOH solution, which afforded compound **8**, with little de-glycosylated side product being generated. After purification by HPLC, the target molecule **8** was obtained as its triethylammonium salt in 68% yield, with **26** recovered in a yield of 15%. The biological activity assay of all the compounds synthesized is underway.

## 3. Experimental 

### 3.1. General

HR-ESI-MS and ESI-MS were performed with a Bruker BIFLEX III instrument. ^1^H-NMR and ^13^C-NMR were recorded with a Bruker AVANCE III 400; CDCl_3_, DMSO-*d*6 or D_2_O were used as a solvent. Chemical shifts are reported in parts per million downfield from TMS (^1^H and ^13^C). ^31^P-NMR spectra were recorded at room temperature by use of a JEOL AL300 spectrometer (121.5 MHz) or JEOL ECA600 spectrometer (243 MHz). Orthophosphoric acid (85%) was used as external standard. ^19^F- NMR spectra were recorded on a Varian VXR-500 spectrometer (470 MHz). Chemical shifts of ^19^F- NMR are reported in ppm with reference to CF_3_COOH as external standard. Compounds **19**, **23**, **26**, and **5**-**8** were purified on an Alltech preparative C_18_ reversed-phase column (2.2 × 25 cm) with a Gilson HPLC using MeCN/TEAB (pH 7.5) buffer system as eluent. 

### 3.2. Synthesis

*N^9^-[(5*′*-Acetoxyethoxy)methyl]-8-bromoadenine* (**10**). To a stirred suspension of 8-bromoadenine (4.5 g, 21.03 mmol) [14] in anhydrous THF (400 mL) was added potassium *tert*-butoxide (2.59 g, 23.13 mmol) and 18-crown-6 (1.11 g, 4.20 mmol). The reaction mixture was stirred at room temperature for 15 min, and then BrCH_2_OCH_2_CH_2_OAc (3.1 mL, 23.13 mmol) [15] was added dropwise at 0 °C. After being stirred for 30 min at 0 °C, the mixture was filtered and the filtrate is evaporated under reduced pressure. The residue was purified by silica gel column chromatography (PE-EA = 1:2) to afford compound **10** (3.02 g, 44%). ^1^H-NMR (400 MHz, DMSO-*d6*) *δ* 1.92 (s, 3H, OAc), 3.69-3.72 (m, 2 H, H_4_′), 4.04-4.17 (m, 2 H, H_5_′), 5.51 (s, 2H, H_1_′), 7.48 (s, 2H, NH_2_), 8.16 (s, 1H, H_2_). ^13^C-NMR (100 MHz, DMSO-*d6*) *δ* 170.1, 154.8, 153.2, 151.2, 126.5, 118.7, 72.3, 67.0, 62.7, 20.5. MS (ESI-TOF^+^): *m/z* = 330.0 [(M + H)^+^].

*N^9^-[(5*′*-Hydroxylethoxy)methyl]-8-bromohypoxanthine* (**12**). Compound **10** (1.43 g, 4.34 mmol) was dissolved in methanol (120 mL). To the solution was added K_2_CO_3_ (73 mg, 0.53 mmol) and stirred for 6 h at room temperature. The mixture was neutralized by addition of 0.1 M HCl solution, and evaporated under reduced pressure*.* The residue was dissolved in AcOH (70 mL), and a solution of NaNO_2_ (2.52 g, 36.4 mmol) in H_2_O (17 mL) was added. The resulting mixture was stirred at room temperature for 24 h. After the mixture was evaporated *in vacuo*, the residue was partitioned between CHCl_3_ and H_2_O. The aqueous phase was extracted again with CHCl_3_, the organic layer was combined and washed with brine, dried (Na_2_SO_4_), filtered and concentrated *in vacuo*. Flash chromatography (CH_2_Cl_2_-MeOH = 40:1) afforded **12** (792 mg, 63% for two steps). ^1^H-NMR (400 MHz, DMSO-*d6*) *δ* 3.46-4.51 (m, 4H, H_4_′, H_5_′), 4.63(s, 1H, OH), 5.32 (s, 2H, H_1_′), 8.14 (s, 1H, H_2_), 12.56 (s, 1H, NH). MS (ESI-TOF^+^): *m/z* = 289.2 [(M + H)^+^].

*N^9^-[(5*′*-tert-Butyldiphenylsilyloxyethoxy)methyl]-8-bromohypoxanthine* (**13**). To a solution of **12** (700 mg, 2.42 mmol) in anhydrous DMF (10 mL) was added imidazole (1.86 g, 24.2 mmol) and *tert*-butyldiphenylsilyl chloride (3.4 mL, 12.1 mmol) under argon, and the mixture was stirred at room temperature for 12 h. And the mixture was evaporated *in vacuo*, the residue was partitioned between CH_2_Cl_2_ and H_2_O. The aqueous phase was extracted again with CH_2_Cl_2_, the organic layer was combined and washed with brine, dried (Na_2_SO_4_), filtered and concentrated *in vacuo*. Flash chromatography (PE-acetone = 5:1) afforded compound **13** (1.21 g, 95%). ^1^H-NMR (400 MHz, CDCl_3_) *δ* 1.06 (s, 9H, (CH_3_)_3_C-), 3.71-3.73 (m, 2H, H_4_′), 3.82-3.84 (m, 2H, H_5_′), 5.66 (s, 2H, H_1_′), 7.37-7.69 (m, 10H, ArH), 8.44 (s, 1H, H_2_),13.19 (s, 1H, NH). ^13^C-NMR (100 MHz, CDCl_3_) *δ* 157.8, 150.8, 146.3, 135.5, 133.3, 129.6, 127.6, 126.4, 124.6, 77.3, 77.0, 76.7, 73.5, 71.1, 62.9, 26.7, 19.0. HRMS (ESI-TOF^+^): calcd for C_24_H_27_BrN_4_O_3_Si [(M + H)^+^] 527.1109, [(M + Na)^+^] 549.0928, [(M + K)^+^] 565.0662; found, 527.1109, 549.0931, 565.0667.

*N^1^-[(5*′′*-Acetoxyethoxy)methyl]-N^9^-[(5*′*-tert-butyldiphenylsilyloxyethoxy)methyl]-8-bromohypoxanthine* (**14**). To the solution of **13** (1.23 g, 2.33 mmol) and DBU (3.5 mL, 23.3 mmol) in anhydrous CH_2_Cl_2_ (25 mL) was added ClCH_2_OCH_2_CH_2_OAc (1.8 mL, 11.65 mmol) [15] dropwise at 0 °C. After being stirred for 40 min at room temperature, the solvent was evaporated *in vacuo* and the residue was purified by silica gel column chromatography (PE-acetone = 5:1) to afford compound **14** (916 mg, 61%). ^1^H-NMR (400 MHz, CDCl_3_) *δ* 1.04 (s, 9H, (CH_3_)_3_C-), 2.03 (s, 3H, OAc), 3.68-3.70 (m, 2H, H_4_′), 3.79-3.82 (m, 2H, H_5_′), 3.85-3.87 (m, 2H, H_4_′′), 4.18-4.20 (m, 2H, H_5_′′), 5.54 (s, 2H, H_1_′′), 5.61 (s, 2H, H_1_′), 7.35-7.66 (m, 10H, ArH), 8.09 (s, 1H, H_2_). ^13^C-NMR (100 MHz, CDCl_3_) *δ* 170.7, 155.3, 149.2, 147.8, 135.5, 133.2, 129.7, 127.6, 126.2, 124.3, 75.0, 73.5, 71.0, 68.1, 62.9, 26.7, 20.7, 19.0. HRMS (ESI-TOF^+^): calcd for C_29_H_35_BrN_4_O_6_Si [(M + H)^+^] 643.1582, [(M + Na)^+^] 665.1401, [(M + K)^+^] 681.1135; found, 643.1563, 665.1377, 681.1117.

*N^1^-[(5*′′*-Acetoxyethoxy)methyl]-N^9^-[(5*′*-tert-butyldiphenylsilyloxyethoxy)methyl]-8-trifluoromethyl- hypoxanthine* (**15**). To a solution of compound **14** (576 mg, 0.895 mmol) and CuI (206 mg, 1.074 mmol) in anhydrous DMF (33 mL), hexamethylphosphoric triamide (2.39 mL, 13.425 mmol) and FSO_2_CF_2_CO_2_Me (1.71 mL, 13.425 mmol) were added successively. The reaction mixture was stirred for 20 h at 70 °C under argon, then cooled to room temperature, 22 mL of saturated aq. NH_4_Cl was added and the mixture was extracted with 200 mL of EA-hexanes (7:3). The organic layer was washed successively with sat. aq. NaHCO_3_, water and brine, dried (Na_2_SO_4_), filtered, and concentrated under reduced pressure. The residue was purified by silica gel column chromatography (PE-acetone = 9:2) to afford compound **15** (238 mg, 42%) and compound **16** (48 mg, 14%), with compound **14** recovered (96 mg, 17%). ^1^H-NMR (400 MHz, CDCl_3_) *δ* 1.03 (s, 9H, (CH_3_)_3_C-), 2.04 (s, 3H, OAc), 3.68-3.70 (m, 2H, H_4_′), 3.79-3.81 (m, 2H, H_5_′), 3.87-3.89 (m, 2H, H_4_′′), 4.20-4.22 (m, 2H, H_5_′′), 5.56 (s, 2H, H_1_′′), 5.76 (s, 2H, H_1_′), 7.35-7.66 (m, 10H, ArH), 8.18 (s, 1H, H_2_). ^13^C-NMR (100 MHz, CDCl_3_) *δ* 170.7, 156.3, 149.4, 149.3, 138.6 (d, ^2^*J*_CF_ = 41 Hz), 135.5, 133.2, 129.7, 127.7, 122.7, 118.2 (q, ^1^*J*_CF_ = 270 Hz), 75.1, 73.5, 71.4, 68.3, 63.1, 63.0, 26.7, 20.8, 19.1. ^19^F-NMR (470 MHz, CDCl_3_) *δ* -63.4 (s). HRMS (ESI-TOF^+^): calcd for C_30_H_35_F_3_N_4_O_6_Si [(M + Na)^+^] 655.2170, [(M + K)^+^] 671.1904; found, 655.2169, 671.1913.

*N^1^-[(5*′′*-Acetoxyethoxy)methyl]-N^9^-[(5*′*-hydroxylethoxy)methyl]-8-trifluoromethylhypoxanthine* (**16**). A solution of **15** (182 mg, 0.288 mmol) in anhydrous THF (35 mL) was added 70% HF·Py 1.3 mL at −20 °C. The mixture was stirred at 0 °C for 1 h and at room temperature over night. The reaction mixture was quenched with saturated aq. NaHCO_3_ at 0 °C and diluted with ethyl acetate, then partitioned and the water layer was washed with ethyl acetate again. The organic layer was combined, washed with brine, dried (Na_2_SO_4_), filtered and concentrated under reduced pressure. The residue was purified by silica gel column chromatography (PE-EA = 1:5) to afford the compound **16** (91 mg, 82%). ^1^H-NMR (400 MHz, CDCl_3_) *δ* 2.05 (s, 3H, OAc), 3.68-3.74 (m, 4H, H_4_′, H_5_′), 3.87-3.89 (m, 2H, H_4_′′), 4.19-4.22 (m, 2H, H_5_′′), 5.56 (s, 2H, H_1_′′), 5.76 (s, 2H, H_1_′), 8.23 (s, 1H, H_2_). ^13^C-NMR (100 MHz, CDCl_3_) *δ* 170.8, 156.2, 149.6, 149.3, 138.4 (q, ^2^*J*_CF_ = 41 Hz), 122.7, 118.2 (q, ^1^*J*_CF_ = 270 Hz), 75.1, 73.2, 71.2, 68.3, 62.9, 61.4, 20.8; ^19^F-NMR (470 MHz, CDCl_3_) *δ*-63.4 (s). HRMS (ESI-TOF^+^): calcd for C_14_H_17_F_3_N_4_O_6_ [(M + Na)^+^] 417.0992, [(M +K)^+^], 433.0726; found, 417.0991, 433.0730.

*N^1^-[(5*′′*-Acetoxyethoxy)methyl]-N^9^-[[5*′*-bis(phenylthio)phosphoryloxyethoxy]-methyl]-8-trifluoro-methylhypoxanthine* (**17**). To a solution of **16** (66 mg, 0.167 mmol) in anhydrous pyridine (5 mL) was added TPSCl (302 mg, 1.00 mmol), PSS (571 mg, 1.50 mmol) [20], and tetrazole (105 mg, 1.50 mmol), and the mixture was stirred at room temperature for 12 h. The mixture was evaporated, and the residue was purified by silica gel column chromatography (PE-EA = 1:2) to give compound **17** (86 mg, 79%). ^1^H NMR (400 MHz, CDCl_3_) *δ* 2.05 (s, 3H, OAc), 3.82-3.84 (m, 2H, H_4_′), 3.86-3.88 (m, 2H, H_5_′), 4.18-4.21 (m, 2H, H_4_′′), 4.31-4.35 (m, 2H, H_5_′′), 5.56 (s, 2H, H_1_′′), 5.68 (s, 2H, H_1_′), 7.33-7.52 (m, 10H, ArH), 8.22 (s, 1H, H_2_). ^13^C-NMR (100 MHz, CDCl_3_) *δ* 170.8, 156.3, 149.7, 149.3, 138.5 (q, ^2^*J*_CF_ = 41 Hz), 135.3, 129.7, 129.4, 125.9, 122.7, 118.2 (q, ^1^*J*_CF_ = 269 Hz), 75.2, 73.0, 69.0, 68.4, 66.2, 62.9, 20.8. ^19^F NMR (470 MHz, CDCl_3_) *δ*-63.4 (s). ^31^P-NMR (D_2_O, 243 MHz, decoupled with ^1^H) *δ*50.41 (s). HRMS (ESI-TOF^+^): calcd for C_30_H_35_F_3_N_4_O_6_Si [(M + H)^+^], 659.1005; found, 659.1006.

*N^1^-[(5*′′*-Phosphonoxyethoxy)methyl]-N^9^-[[5*′*-(phenylthio)phosphoryloxyethoxy]methyl]-8-trifluoro- methylhypoxanthine* (**19**). Compound **17** (54 mg, 0.082 mmol) was dissolved in MeOH (4 mL), and a solution of acetyl chloride (7 μL, 0.098 mmol) in anhydrous CH_2_Cl_2_ (1 mL) was added at −20 °C. The mixture was stirred at 0 °C for 30 min and raised to room temperature for 24 h, then neutralized by sat. aq. NaHCO_3_ solution. The mixture was evaporated, and the residue was partitioned between CH_2_Cl_2_ and H_2_O. The aqueous phase was extracted again with CH_2_Cl_2_, the organic layers were combined and washed with brine, dried (Na_2_SO_4_), filtered and concentrated *in vacuo*. The residue was purified by silica gel column chromatography (PE-EA =1:10) to give compound **18** (31 mg). The deacetylated product **18** (31 mg, 0.050 mmol) was dissolved in anhydrous CH_3_CN (8 mL). DIPEA (65 μL, 0.375 mmol) and POCl_3_ (28 μL, 0.300 mmol) were added successively to the solution at −20 °C, and the mixture was stirred at 0 °C for 14 h, and then added 5mL of TEAB (1 M, pH 7.5) at 0 °C and stirred at room temperature for 6 h. After evaporation under reduced pressure, the residue was partitioned between H_2_O and CHCl_3_, and the aqueous layer was washed with CHCl_3_ and evaporated *in vacuo*. The residue was dissolved in 5 mL of TEAB buffer (0.05 M, pH 7.5), then applied to a C_18_ reversed-phase column (2.2 × 25 cm). The column was developed using a linear gradient of 0-40% CH_3_CN in TEAB buffer (0.05 M, pH 7.5) within 30 min to afford **19** (27 mg, 41% for two steps) as its triethylammonium salt. ^1^H-NMR (400 MHz, D_2_O) *δ* 3.70-3.73 (m, 4H, H_4_′, H_5_′), 3.85-3.89 (m, 2H, H_4_′′), 3.94-3.98 (m, 2H, H_5_′′), 5.45 (s, 2H, H_1_′′), 5.64 (s, 2H, H_1_′), 7.09-7.29 (m, 5H, ArH), 8.41 (s, 1H, H_2_). ^13^C-NMR (100 MHz, D_2_O) *δ* 157.6, 150.9, 149.5, 138.8 (q, ^2^*J*_CF_ = 41 Hz), 132.7, 129.6, 128.9, 127.7, 122.1, 117.8 (q, ^1^*J*_CF_ = 270 Hz), 76.4, 73.3, 69.3, 64.9, 64.3, 46.6, 8.2. ^19^F-NMR (470 MHz, D_2_O) *δ*-63.0 (s). ^31^P-NMR (D_2_O, 243 MHz, decoupled with ^1^H) *δ* 1.10 (s), 17.80 (s). HRMS (ESI-TOF^−^) calcd for C_18_H_21_N_4_O_10_P_2_SF_3_ [(M − H)^−^], 603.0333; found, 603.0331.

*N^1^-[(5*′′*-O-Phosphorylethoxy)methyl]-N^9^-[(5*′*-O-phosphorylethoxy)methyl]-8-trifluoromethylhypo- xanthine-cyclic pyrophosphate* (**5**). A solution of **19** (5 mg, 6.1 μmol) in anhydrous pyridine (4.5 mL) was added slowly over 20 h, utilizing a syringe pump, to a mixture of I_2_ (36 mg, 142 μmol) and 3 Å molecular sieves (0.36 g), in pyridine (40 mL) at room temperature in the dark. The molecular sieves were filtered off with Celite and washed with H_2_O. The combined filtrate was evaporated, and the residue was partitioned between CHCl_3_ and H_2_O. The aqueous layer was evaporated, and the residue was dissolved in 0.05 M TEAB buffer, which was applied to C_18_ reversed-phase column (2.2 × 25 cm). The column was developed using a linear gradient of 0-20% CH_3_CN in TEAB buffer (0.05 M, pH 7.5) within 30 min to give **5** as its triethylammonium salt (3.0 mg, 71%). ^1^H-NMR (400 MHz, D_2_O) *δ* 3.70-3.78 (m, 4H, H_4_′, H_5_′), 3.81-3.83 (m, 2H, H_4_′′), 3.88-3.90 (m, 2H, H_5_′′), 5.54 (s, 2H, H_1_′′), 5.75 (s, 2H, H_1_′), 8.49 (s, 1H, H_2_). ^19^F-NMR (470 MHz, D_2_O) *δ*-62.5 (s). ^31^P-NMR (D_2_O 121.5 MHz, decoupled with ^1^H) *δ* −10.07 (d, *J*_P,P_ = 18.2 Hz), −10.42 (d, *J*_P,P_ = 18.2 Hz). HRMS (ESI-TOF^−^) calcd for C_12_H_15_N_4_O_10_P_2_F_3_ [(M − H)^−^], 493.0143; found, 493.0146.

*N^1^-[(5*′′*-Phosphonoxyethoxy)methyl]-N^9^-[(5*′*-Phosphonoxyethoxy)methyl]-8- trifluoromethylinosine* (**6**). Compound **16** (20 mg, 0.051 mmol) was dissolved in methanol (2 mL). To the solution was added K_2_CO_3_ (1 mg, 7.24 μmol) at room temperature and stirred for 6h. The mixture was neutralized by addition of 0.01 M HCl solution, and removed of the solvent *in vacuo.* The residue was partitioned between CHCl_3_ and H_2_O, and the organic layer was washed with brine, dried (Na_2_SO4), and evaporated, affording compound **20** (16 mg). Compound **20** (16 mg, 0.045 mmol) was dissolved in anhydrous CH_3_CN (5 mL). DIPEA (94 μL, 0.54 mmol) and POCl_3_ (42 μL, 0.45 mmol) were added successively to the solution at −20 °C. The mixture was stirred at 0 °C for 16 h, and then added 5 mL of TEAB (1 M, pH 7.5) at 0 °C and stirred for 6 h at room temperature. After evaporation under reduced pressure, the residue was partitioned between H_2_O and CHCl_3_, and the aqueous layer was washed with CHCl_3_ and evaporated *in vacuo*. The residue was dissolved in 5 mL of TEAB buffer (0.05 M, pH 7.5), and applied to a C_18_ reversed-phase column (2.2 × 25 cm)**.** The column was developed using a linear gradient of 0-40% CH_3_CN in TEAB buffer (0.05 M, pH 7.5) within 30 min to give **6** (22.3 mg, 62% for two steps) as its triethylammonium salt. ^1^H-NMR (400 MHz, D_2_O) *δ* 3.73-3.79 (m, 4H, H_4_′, H_5_′), 3.85-3.92 (m, 4H, H_4_′′, H_5_′′), 5.45 (s, 2H, H_1_′′), 5.77 (s, 2H, H_1_′), 8.51 (s, 1H, H_2_). ^13^C-NMR (100 MHz, D_2_O) *δ* 157.8, 151.0, 149.7, 138.6 (q, ^2^*J*_CF_ = 41 Hz), 122.2, 117.9 (q, ^1^*J*_CF_ = 270 Hz), 76.4, 73.3, 69.5, 69.2, 64.1, 63.8. ^19^F-NMR (470 MHz, D_2_O) *δ* -62.9 (s). ^31^P-NMR (D_2_O, 243 MHz, decoupled with ^1^H) *δ* 0.19 (s), 0.22 (s). HRMS (ESI-TOF^−^) calcd for C_12_H_17_N_4_O_11_P_2_F_3_ [(M − H)^−^], 511.0248; found, 511.0246.

*N^1^-[(5*′′*-Phosphonoxyethoxy)methyl]-5*′*-O-phosphoryl-2*′*,3*′*-O-isopropylidene-inosine*(**23**). Compound **21** (49 mg, 0.116 mmol) [6] was dissolved in 24 mL of methanol. To the solution was added K_2_CO_3_ (2 mg, 14.5 μmol) and stirred at room temperature for 6h. The mixture was neutralized by addition of 0.1 M HCl solution, and removed of the solvent *in vacuo.* The residue was partitioned between CHCl_3_ and H_2_O, and the organic layer was washed with brine, dried (Na_2_SO_4_), and evaporated, affording compound **22** (38mg)**.** Compound **22** (38 mg, 0.099 mmol) was dissolved in anhydrous CH_3_CN (5 mL). DIPEA (0.21 mL, 1.19 mmol) and POCl_3_ (91 μL, 0.99 mmol) were added successively to the solution at −20 °C. The mixture was stirred at 0 °C for 16 h, and then was neutralized by addition of 1 M NaOH solution. And the resulting mixture was stirred at room temperature for 2 h. After evaporated under reduced pressure, the residue was partitioned between H_2_O and CHCl_3_, and the aqueous layer was washed with CHCl_3_ and evaporated *in vacuo*. The residue was dissolved in 5 mL of TEAB buffer (0.05 M, pH 7.5), and applied to a C_18_ reversed-phase column (2.2 × 25 cm)**.** The column was developed using a linear gradient of 0-40% CH_3_CN in TEAB buffer (0.05 M, pH 7.5) within 30 min to give **23** (61 mg, 71% for two steps) as its triethylammonium salt. ^1^H-NMR (400 MHz, D_2_O) *δ* 1.31, 1.53 (each s, each 3H, 2 × CH_3_), 3.72-3.74 (m, 2H, H_5_′), 3.85-3.89 (m, 2H, CH_2_O), 3.91-3.94 (m, 2H, CH_2_OP), 4.52-4.56 (m, 1H, H_4_′), 5.06 (dd, 1H, *J*_H3′,H4′_ = 1.6 Hz, *J*_H3′, H2′_ = 6.0 Hz, H_3_′), 5.29 (dd, 1H, *J*_H2′,H1′_= 2.8 Hz, *J*_H2′,H3′_ = 6.0 Hz, H_2_′), 5.47 (d, 1H, *J*_H1″b, H1″a_ = 10.8 Hz, H_1″b_), 5.51 (d, 1H, *J*_H1″a, H1″b_ = 10.8 Hz, H_1″a_), 6.17 (d, 1H, *J*_H1′,H2′_ = 2.8 Hz, H_1_′), 8.23, 8.34 (each s, each 1H, H_8_, H_2_). ^31^P-NMR (D_2_O, 121.5 MHz, decoupled with ^1^H) *δ* 1.79 (s), 1.91 (s). HRMS(ESI-TOF^−^): calcd for C_14_H_24_N_4_O_13_P_2_ [(M − H)^−^], 541.0742; found, 541.0733.

*N^1^-[(5′′-Phosphonoxyethoxy)methyl]-5*′*-O-phosphorylinosine* (**7**). A solution of **23** (25 mg, 33.6 μmol) in 60% HCOOH (6 mL) was stirred for 8 h, and then 14 mL of TEAB (1M, pH 7.5) was added. The solution was evaporated under reduced pressure. The residue was dissolved in 0.05 M TEAB buffer (4.0 mL), which was applied to C_18_ reversed-phase column (2.2 × 25 cm). The column was developed using a linear gradient of 0-40% CH_3_CN in TEAB buffer (0.05 M, pH 7.5) within 30 min to afford **7** as its triethylammonium salt (20.2 mg. 85%). ^1^H-NMR (400 MHz, D_2_O) *δ* 3.72-3.74 (m, 2H, H_5′_), 3.85-3.88 (m, 2H, CH_2_O), 3.98-4.01 (m, 2H, CH_2_OP), 4.23-4.26 (m, 1H, H_4′_), 4.34-4.37 (m, 1H, H_3′_), 4.59-4.61 (m, 1H, H_2′_), 5.46-5.52 (m, 2H, H_1″_), 6.01 (d, 1H, *J*_H1′,H2′_ = 5.6 Hz, H_1′_), 8.31, 8.36 (each s, each 1H, H_8_, H_2_). ^31^P-NMR (D_2_O, 243 MHz, decoupled with ^1^H) *δ* 0.81 (s), 0.92 (s). HRMS (ESI-TOF^−^): calcd for C_14_H_24_N_4_O_13_P_2_ [(M − H)^−^], 501.0429; found, 501.0426.

*N^1^-[(5′′-Phosphonoxyethoxy)methyl]-5′-O-phosphoryl-2*′*,3*′*-O-isopropylidene-8-trifluoromethylinosine* (**26**). By a similar procedure that described for **6**, **26** was synthesized from **24** [9], as its triethylammonium salt, in 57% yield for two steps. ^1^H-NMR (400 MHz, D_2_O) *δ* 1.29, 1.50 (each s, each 3H, 2×CH_3_), 3.71-3.73 (m, 2H, H_5′_), 3.82-3.96 (m, 4H, CH_2_O, CH_2_OP), 4.35-4.39 (m, 1H, H_4′_), 5.19 (dd, 1H, *J*_H3′,H4′_ = 4.0 Hz, *J*_H3′,H2′_ = 6.8 Hz, H_3′_), 5.40 (d, 1H, *J*_H1″a,H1″b_=10.8 Hz, H_1″a_), 5.55-5.60 (m, 2H, H_1″b_, H_2′_), 6.23 (d, 1H, *J*_H1′,H2′_ = 2.0 Hz, H_1′_), 8.44 (s, 1H, H_2_). ^13^C-NMR (100 MHz, D_2_O) *δ* 157.8, 150.6, 148.9, 138.3 (q, ^2^*J*_CF_ = 40Hz), 122.9, 117.8 (q, ^1^*J*_CF_ *=* 270 Hz), 115.4, 90.0, 86.7, 86.6, 83.7, 81.1, 76.4, 69.2, 64.2, 63.8, 25.9, 24.2. ^19^F-NMR (470 MHz, D_2_O) *δ* -61.9 (s). ^31^P-NMR (D_2_O, 243 MHz, decoupled with ^1^H) *δ* 0.54 (s), 0.70 (s). HRMS(ESI-TOF^−^): calcd for C_17_H_23_F_3_N_4_O_13_P_2_ [(M − H)^−^], 609.0612; found, 609.0615. 

*N^1^-[(5′′-Phosphonoxyethoxy)methyl]-5*′*-O-phosphoryl-8-trifluoromethylinosine* (**8**). A solution of **26** (15 mg, 18.47 μmol) in 10% HCOOH (7.5 mL) was stirred at room for 60 h, and then 11 mL of TEAB (1 M, pH 7.5) was added. The solution was evaporated *in vacuo*. The residue was dissolved in 0.05 M TEAB buffer (2.0 mL), which was applied to C_18_ reversed-phase column (2.2 cm × 25 cm). The column was developed using a linear gradient of 0-40% CH_3_CN in TEAB buffer (0.05 M, pH 7.5) within 30 min to afford compound **8** (9.7 mg, 68%) as its triethylammonium salt, with the compound **26** (2.2 mg, 15%) recovered. ^1^H-NMR (400 MHz, D_2_O) *δ* 3.76-3.78 (m, 2H, H_5′_), 3.87-3.91 (m, 2H, CH_2_O), 4.02-4.13 (m, 2H, CH_2_OP), 4.21-4.25 (m, 1H, H_4′_), 4.55-4.57 (m, 1H, H_3′_), 5.20-5.23 (m, 1H, H_2′_), 5.46 (d, 1H, *J*_H1″b, H1″a_ = 10.6 Hz,H_1′b_), 5.59 (d, 1H, *J*_H1″a, H1″b_ = 10.6 Hz, H_1″a_), 5.99 (d, 1H, *J*_H1″,H2′_ = 5.6 Hz, H_1′_), 8.47 (s, 1H, H_2_). ^13^C-NMR (100 MHz, D_2_O) *δ* 157.9, 150.3, 149.4, 138.9 (q, ^2^*J*_CF_ = 40Hz), 123.2, 117.8 (q, ^1^*J*_CF_ *=* 269 Hz), 89.8, 84.4, 76.3, 72.0, 70.0, 69.3, 64.4, 64.1. ^19^F-NMR (470 MHz, D_2_O) *δ* -61.7 (s). ^31^P-NMR (D_2_O, 121.5 MHz, decoupled with ^1^H) *δ* 6.25(s), 6.27 (s). HRMS(ESI-TOF^−^): calcd for C_14_H_19_F_3_N_4_O_13_P_2_ [(M − H)^−^], 569.0303; found, 569.0315. 

## 4. Conclusion

In conclusion, we have successfully synthesized 8-CF_3_-cIDPDE (**5**) *via* construction of *N^1^, N^9^*-disubstituted hypoxanthine, trifluoromethylation and intramolecular condensation. A series of novel acyclic analogues of cADPR, compounds **6**-**8**, were also synthesized by concise synthetic routes. With the special properties of trifluoromethyl, 8-CF_3_-cIDPDE and the acyclic derivatives are expected to provide useful agents to explore the cADPR/RyR Ca^2+^ signalling system and illuminate the structure-activity relationship of cADPR analogues.

## Figures and Tables

**Table 1:** Optimization of the reaction conditions of trifluoromethylation.

Entry	FSO_2_CF_2_CO_2_Me /HMPA	Yield
1	5 equiv	trace
2	10 equiv	12%
3	15 equiv	42%
4	20 equiv	31%
5	30 equiv	18%
